# Advancing Cell-Instructive Biomaterials Through Increased Understanding of Cell Receptor Spacing and Material Surface Functionalization

**DOI:** 10.1007/s40883-020-00180-0

**Published:** 2020-11-20

**Authors:** Stephanie A. Maynard, Charles W. Winter, Eoghan M. Cunnane, Molly M. Stevens

**Affiliations:** Department of Materials, Department of Bioengineering and Institute of Biomedical Engineering, Imperial College London, London, SW7 2AZ UK

**Keywords:** Bio-mimicry, Bio-instructive, Biomaterials, Nanoscale ligand spacing, Functionalization, Cell adhesion, Integrins, Receptor clustering

## Abstract

**Abstract:**

Regenerative medicine is aimed at restoring normal tissue function and can benefit from the application of tissue engineering and nano-therapeutics. In order for regenerative therapies to be effective, the spatiotemporal integration of tissue-engineered scaffolds by the native tissue, and the binding/release of therapeutic payloads by nano-materials, must be tightly controlled at the nanoscale in order to direct cell fate. However, due to a lack of insight regarding cell–material interactions at the nanoscale and subsequent downstream signaling, the clinical translation of regenerative therapies is limited due to poor material integration, rapid clearance, and complications such as graft-versus-host disease. This review paper is intended to outline our current understanding of cell–material interactions with the aim of highlighting potential areas for knowledge advancement or application in the field of regenerative medicine. This is achieved by reviewing the nanoscale organization of key cell surface receptors, the current techniques used to control the presentation of cell-interactive molecules on material surfaces, and the most advanced techniques for characterizing the interactions that occur between cell surface receptors and materials intended for use in regenerative medicine.

**Lay Summary:**

The combination of biology, chemistry, materials science, and imaging technology affords exciting opportunities to better diagnose and treat a wide range of diseases. Recent advances in imaging technologies have enabled better understanding of the specific interactions that occur between human cells and their immediate surroundings in both health and disease. This biological understanding can be used to design smart therapies and tissue replacements that better mimic native tissue. Here, we discuss the advances in molecular biology and technologies that can be employed to functionalize materials and characterize their interaction with biological entities to facilitate the design of more sophisticated medical therapies.

## Introduction

The goal of regenerative medicine is to restore normal tissue function by combining molecular biology and material science [1]. The translational research that underpins regenerative medicine often employs a “biomaterial” that is implanted to actively augment existing biological processes and facilitate repair. Such biomaterials range in scale from tissue-engineered scaffolds intended for whole organ replacement to nano-materials intended for targeted therapeutic drug delivery. In order for regenerative therapies to be effective, the spatiotemporal integration of tissue-engineered scaffolds by the native tissue, and the binding/release of therapeutic payloads by nano-materials, must be tightly controlled. However, due to a lack of insight regarding cell–material signaling interactions at the nanoscale, the majority of implanted biomaterials are either rejected by the host or rapidly cleared from the tissue, thus limiting the current clinical translation status of many regenerative therapies [2]. This review is intended to aid in directing therapies toward effective regenerative outcomes by outlining the current understanding of nanoscale cell–material interactions. Specifically, we review the organization of key cell surface receptors and the current fabrication techniques used to control the presentation of cell-interactive molecules on biomaterial surfaces, as well as presenting several important and advanced techniques for characterizing the interactions that occur between cell surface receptors and biomaterials intended for use in regenerative medicine.

## Nanoscale Cell Surface Receptor Regulation for Bio-instructive Therapeutic Design

**Fig. 1:**
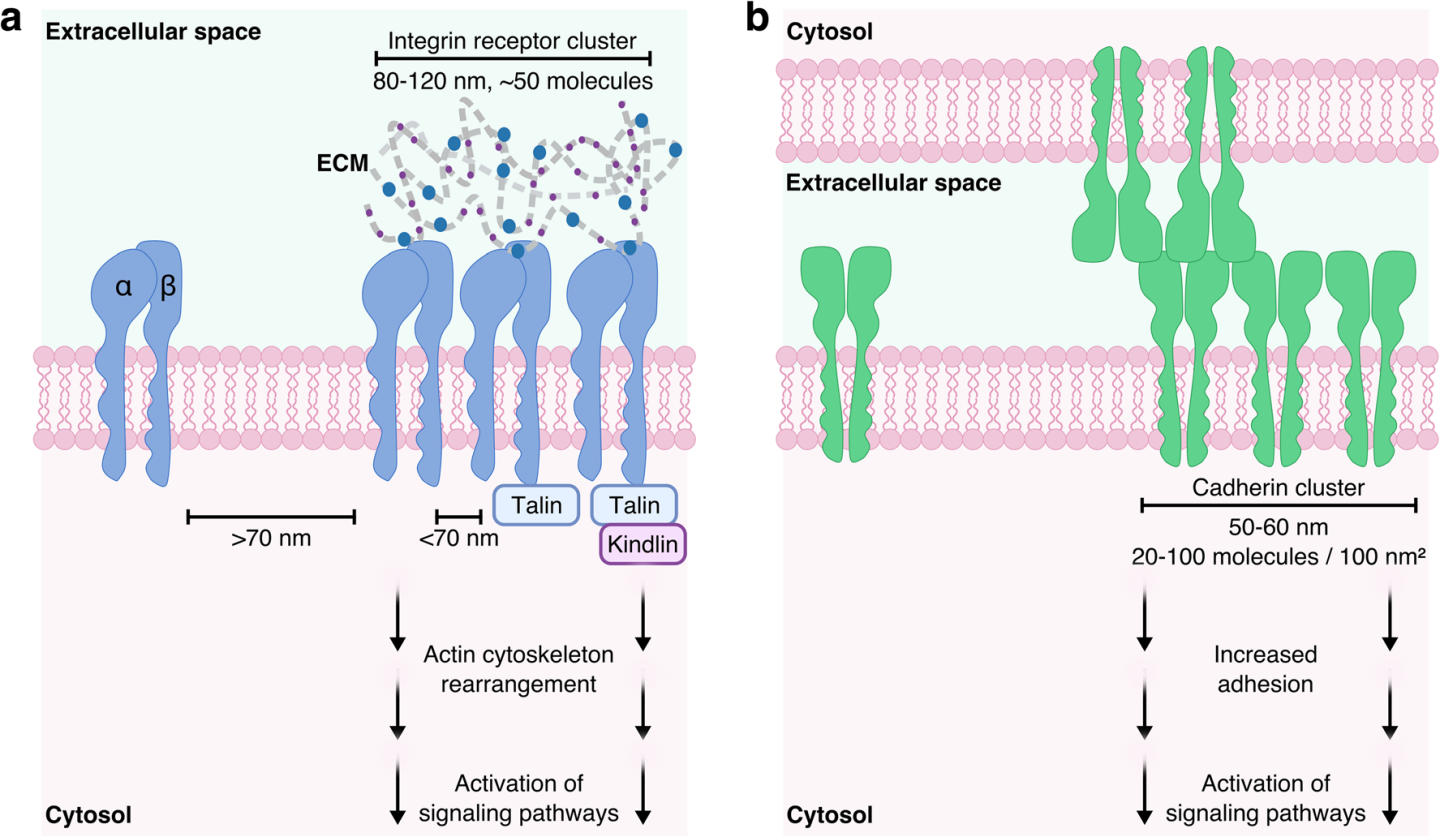
Cell surface adhesion receptor spatial presentation. **a** Integrin clustering occurs when integrins are closer than 70 nm. Integrin receptor clusters are highly controlled, with cluster sizes found conserved at 80–120 nm in diameter and containing around 50 molecules. Integrins additionally bind to intracellular adaptor proteins, such as talin and kindlin, further stabilizing the clusters. These adaptor proteins additionally bind the actin cytoskeleton, transmitting the forces generated by integrins that bind to the extracellular matrix (ECM) into migratory cell behavior. **b** Cadherin cluster size is highly conserved at around 50–60 nm in diameter; however, molecular density varies in range from 20 - 100 molecules per 100 nm^2^. Cadherins form *cis*-homodimers, which laterally associate into clusters and *trans*-dimerize with cadherins in neighboring cell membranes forming cell–cell adhesions

Complex biophysical regulation of cell signaling occurs at the nanoscale and governs the processes of tissue development, maturation, homeostasis, and repair. Physical and chemical stimuli from other cells, the extracellular matrix (ECM), or soluble signaling molecules cause specific, controlled downstream signaling cascades. In this way, cells can both sense environmental cues and respond by modifying their behavior, or altering the synthesis/breakdown of ECM in their immediate surroundings. Errors in signaling and processing of cellular information can result in disease, with cells no longer able to control their microenvironment or react to pathological changes. Recent technological advances, such as those in super-resolution microscopy (see the “Nanoscale Imaging of Cell Surface Receptor Organization and Cell–Material Interactions” section), now enable visualization and quantification of cell surface receptor number, clustering, and subsequent signaling at the nanoscale. Adhesion receptors enable cells to bind, sense, and respond to their environment through nanoscale organization of their surface presentation. There are four adhesion receptor superfamilies: integrins, cadherins, selectins, and immunoglobulin (Ig) cell adhesion molecules (CAMs). Here, we focus on integrins (the major cell–matrix adhesion receptors) and cadherins (prevalent cell–cell adhesion receptors) (Fig. 1), and how they provide important targets in regenerative medicine to ensure that the interactions between implanted biomaterials and the surrounding cells lead to effective regenerative outcomes.

### Role of Integrins in Health, Disease, and Regenerative Medicine

Integrins are a superfamily of 24 known transmembrane heterodimeric adhesion receptors, formed from the non-covalent interaction between an *alpha* (18 subtypes) and a *beta* (8 subtypes) subunit [3], and are around 12 nm in size [4]. They are involved in integrating chemical and mechanical signals in a bidirectional manner across the plasma membrane, enabling cells to sense and respond to their extracellular environment through coordination with intracellular pathways [5]. Integrins are almost ubiquitously expressed across all cell types and bind a wide range of ligands, with a number of integrins capable of binding the same ligand [6]. Specifically, most integrins bind components of the ECM, such as collagen (α1β1, α2β1, α10β1, α11β1) [7], laminin (α3β1, α6β1, α6β4, α7β1) [8], and fibronectin (α5β1, αvβ3) [4, 9], making them vital for cell adhesion and migration. The conformation of the integrin receptor provides further regulation of ligand binding, with inactive integrins assuming a bent conformation. Inactive integrins can then be activated through force generation or intracellular biochemical interactions, leading to an extended conformation which induces an increase in affinity for its ligand and strong adhesion to the ECM [10]. Importantly, additional signaling mechanisms arise due to integrin clustering at the plasma membrane following ligand binding.

Using a rigid template of gold nanodots functionalized with the binding peptide arginyl-glycyl-aspartic acid (RGD), it was demonstrated that cells recognize integrins as being clustered when the receptors are less than 70 nm apart [11]. This clustering directly influenced cell adhesion and spreading on the substrate. Despite the large number of different integrins and variation in their ligands and biological purpose, individual integrin clusters have been shown to remain around 80–120 nm in diameter (Fig. 1a) and contain around 50 molecules [12]. This conserved integrin cluster size was exhibited by cells interfacing with substrates of widely varying rigidities, and thus can be considered a mechanism of cell adhesion applicable to most tissues of the body. Integrin clusters form a key component of the cellular adhesome [13], ranging from early nascent adhesions, with < 1 μm assemblies of clusters of active integrins, to mature focal adhesions, 1–5 μm in size, linking the ECM to the cell cytoskeleton via a complex of intracellular adaptor proteins, including talins and kindlins [14–17]. Constant recycling and endocytosis of integrins at the membrane facilitate cell migration through generation of new adhesion sites of active integrin clusters [18].

Integrin clustering is crucial to the correct functioning of immune cells in healthy tissue. Leukocyte-specific integrins are specialized for immune regulatory functions and tissue repair [19–21]. For example, the T cell–specific integrin, αLβ2 (LFA-1), is activated at sites of inflammation and induces its extended conformation to enable the T cell to bind and cross the endothelium and reach sites of injury [21–23]. It was understood that clustering of αLβ2 at the immune synapse enables communication between T cells and antigen-presenting cells (APCs) [24], but the signaling mechanisms remained unclear. Consequently, various material approaches were employed to further understand the effect of ligand spatial presentation on integrin clustering of T cells and APCs. These include the use of micropatterning of costimulatory ligands [25], supported lipid bilayers presenting tethered proteins [26], and biofunctionalized gold nanoarrays [27]. The strength of T cell response on the CD-3-functionalized gold nanoarrays decreased with increased ligand spacing, with 69 nm spacing generating only a background T cell response [27]. Thus, ligand spacing at the nanoscale can dramatically affect the immune response, an effect important to consider when introducing biomaterials to the body as potential therapies.

Due to the role of integrins in cell adhesion and migration, numerous integrins are implicated in pathologies such as infection, inflammation, and cancers. Infectious agents have been shown to exploit integrin binding to enable their cellular internalization, while changes in integrin presentation and clustering are known to be implicated in virus and bacterial entry into cells. *Staphylococcus aureus* binds fibronectin which mediates an interaction with integrin αIIbβ3 [28], Papilloma virus binds α6β4 directly [29], and Ebola virus binds α5β1 [30]. Blocking the interaction between specific viruses and integrin clusters on the host cells is being explored as a promising route to anti-viral therapies. Tumor growth and invasion partially involve ECM binding [31], and several integrins have been identified as key players in carcinogenesis of tissues throughout the body, including α5β1 [32–34] and αvβ3 [35].

Many nano-therapeutics are therefore aimed toward blocking integrin signaling. PEGylated titanium dioxide nanoparticles were shown to inhibit cancer cell migration via decreasing the cell surface expression of β1 integrins [36]. Gold nanoparticles targeted to integrin αvβ3 inhibited the integrin-dependent melanoma tumor cell adhesion to vitronectin, with very low non-specific background binding, making them a highly selective diagnostic probe and therapy [37]. Recently, a study quantified the density of integrin αvβ3 on glioblastoma cells and found that the density of receptors dictated the cell response to inhibitor molecules [38]. Cell viability and invasion following the inhibitor treatment correlated with the density of the integrin receptor at the surface.

A handful of tissue engineering studies have tried to incorporate the precise positioning of ligands into their material designs to better understand how ligand density can affect cell–material interactions, particularly through integrin clustering. Functionalizing titanium implants with polymer brushes coated with clusters of different fibronectin domains improved implant–tissue integration in vivo through enhanced integrin α5β1 clustering and binding [39]. The interplay between spacing of RGD within nanoarrays (46 and 95 nm) and the size of the arrays (35 and 65 μm length) showed a complex relationship between the length scales on differentiation of mesenchymal stem cells (MSCs) down adipogenic and osteogenic lineages [40]. Using elastin-like electrospun fabric, one study showed clustering of ligands enhanced integrin-dependent clustering and subsequent signaling as a function of the global ligand density [41]. Furthermore, they determined that clustered ligands enhanced cell proliferation and increased the number of focal adhesions. Most recently, integrin-specific hydrogels enhanced the survival and osteo-reparative functions of MSCs by modulating their cytokine production and gene expression of factors associated with bone formation and immunomodulation [42]. Much remains to be understood regarding the downstream signaling of clustered integrins and regulation of receptor availability at the cell surface. However, cell–material interaction studies have enabled a better understanding of how integrin clustering at the nanoscale affects cell behavior at the microscale. Future studies should incorporate precise ligand positioning on the material surface to ensure that biomaterials intended for regenerative medicine applications are integrated effectively into the host.

### Role of Cadherins in Health, Disease, and Regenerative Medicine

Cadherins are a superfamily of membrane-spanning adhesion molecules, formed of homodimers with the extracellular portion measuring 20 nm in length [43], which associate into macromolecular complexes at the cell surface. In humans, over 80 different types of cadherins have been sequenced [44]. Cadherins are present in almost all cell types and are involved in cell–cell junctions, cell polarity, and hence structural integrity of tissues [45]. There are several cadherin subtypes, classified primarily by the location in which they are found, e.g., neural (N)-cadherin and epithelial (E)-cadherin [46]. The biological function of cadherins is regulated at the molecular level via their organization into lateral clusters at the cell surface, which are distributed extensively throughout cell–cell junctions. Cadherin nanoclusters have been demonstrated, using super-resolution microscopy, to maintain a diameter of 50–60 nm (Fig. 1b), with cadherin molecule densities varying in magnitude between 20 and 100/100 nm^2^ [47–49]. Larger microclusters of 1–2 μm form from aggregates of ligand-bound nanoclusters [50]. The cell–cell adhesion of cadherins is driven by *cis*/*trans* dimerization of the homomers [43, 51]. Cadherins in the same plasma membrane form *cis* dimers (parallel), which interact with cadherins in the plasma membrane of the adjacent cell to form *trans* dimers [52]. Accordingly, close spatial presentation of cadherins is implicated in their effective function. The clustering of cadherins is believed to strengthen adhesion, with adhesive strength correlating with the number of microclusters [50, 53]. Lateral clustering provides a signaling hub through interactions with other proteins, such as catenins, as well as a mechanical link to the actin cytoskeleton [54]. Using colloidal lithography, a threshold of 173 nm diameter patterns was estimated to be necessary for epithelial cell attachment to E-cadherin [55]. In order to understand the relationship between receptor density and the adhesive forces of cadherins, one study used self-assembled monolayers of thiols, to which they bound extracellular fragments of E-cadherin, and measured cell binding using single-molecule force spectroscopy (SMFS) [56]. They found that a lateral distance of 5–11 nm was optimal for E-cadherin function.

Similar to integrins, cadherins are involved in cellular migration, immune surveillance, and wound healing. During development, cadherins assist in the positioning of cells [57–59] through a process termed epithelial-to-mesenchymal transition (EMT), whereby epithelial cells lose their cell–cell connections (through regulated decreased expression of cadherins), reorganize their cytoskeleton, and acquire migratory behavior [60]. However, EMT is also a mechanism associated with pathologies involving dysregulation of wound healing. This includes fibrosis [61] and cancer [62]. Fibrosis is a major hurdle in regenerative medicine, as introducing foreign materials into the body can induce fibrosis, negatively impacting the biochemical and mechanical properties of the regenerated tissue [63, 64]. Recent studies using materials aimed at clustering cadherins are exploring their use in improved tissue implantation without inducing fibrosis. EMT causes significant problems for the use of vascular implants, such as stents, with stiff substrates causing endothelial cells to lose their phenotype and undergo EMT. This phenomenon was studied on poly-L-lysine/hyaluronate acid multilayer films with controlled stiffness [65]. Cadherin mimetic peptides immobilized on material surfaces were shown to induce increased cadherin surface expression and clustering which in turn increased epithelial cell adhesion [66]. Percutaneous titanium implants, functionalized with E-cadherin, demonstrated increased epidermal adhesion with limited fibroblast attachment, thereby providing a promising approach to skin grafts with improved implant integration and decreased fibrotic scarring [67]. The EMT phenotype is associated with invasion and migration of cancer cells, and material properties are now being studied for their effect on tissue stiffness and the induction of EMT phenotypes as a tool to study tumorigenesis as well as a basis for chemotherapeutics [65, 68, 69]. Chitosan–hyaluronan membranes were used to study 3D tumor spheroids and found hyaluronan concentration scaled with increasing tumor size and higher EMT phenotype, including increased expression of cadherins and tumor invasiveness [68]. Nanoparticle-based delivery systems aimed at upregulation of E-cadherin are thought to be a promising approach to inhibiting the progression of certain cancers. Unmodified gold nanoparticles were shown to upregulate E-cadherin expression and reverse EMT, thereby inhibiting tumor growth in two models of ovarian cancer [70].

Biomaterial exploitation of cadherin clustering, and therefore the functioning of cell–cell adhesions, is less widely investigated compared with integrin clustering, and research in this area is likely to increase in the coming years. The information presented in the preceding section, regarding how spatial presentation of ligands influences cell fate, could be readily employed to overcome current problems facing the clinical translation of biomaterials and nano-materials, such as rejection by the host or rapid clearance from the tissue.

## Material-Based Techniques for Studying Cell Surface Receptors and Fabricating Cell-Instructive Biomaterials

**Fig. 2:**
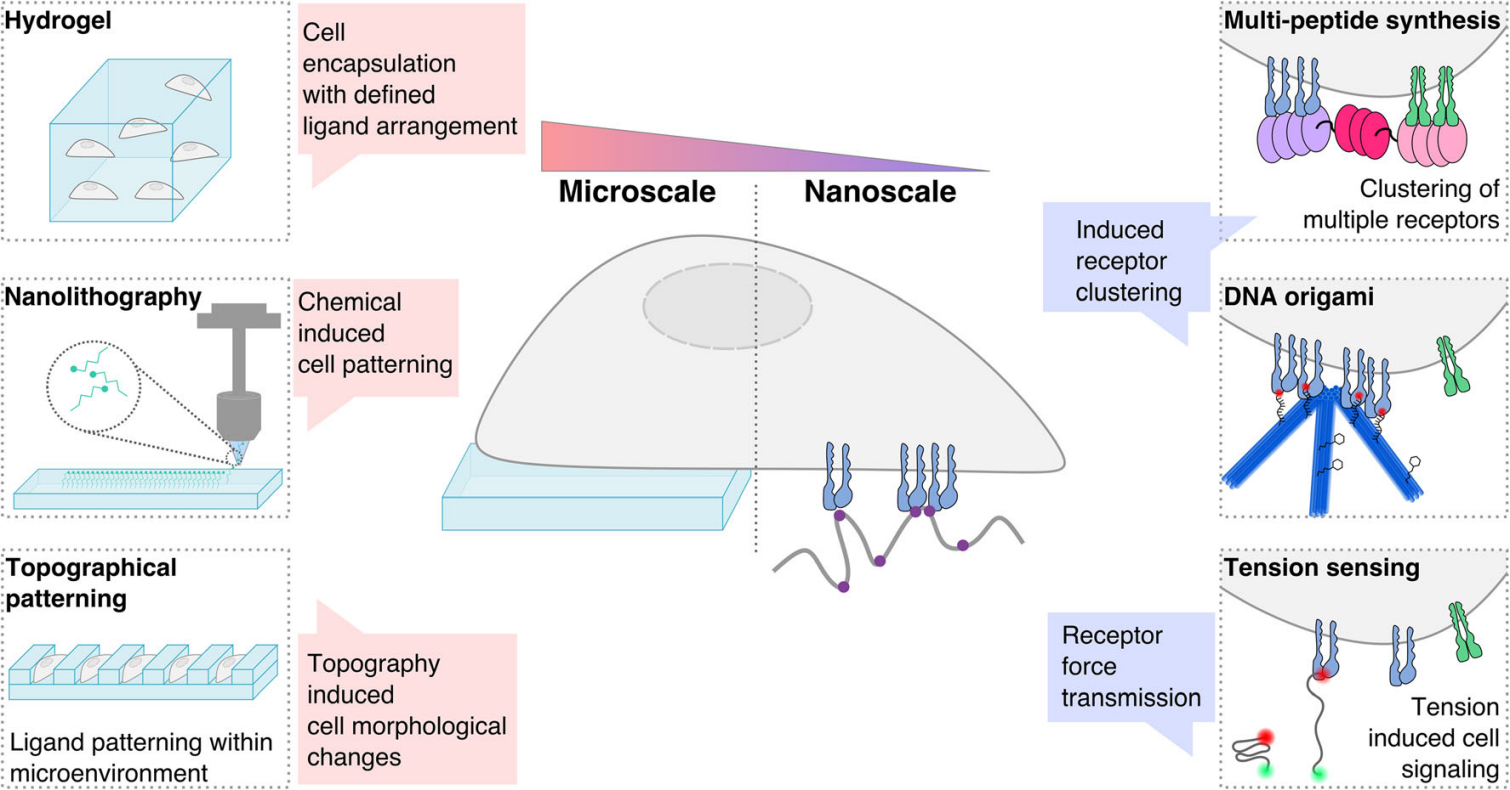
Material techniques for studying cell–material interactions. Fabrication techniques range from the micro- to the nanoscale, thereby influencing cell fate across multiple length scales. Cells can be encapsulated in hydrogels at the macroscale, with ligands arranged for cell binding. Nanolithography enables precise patterning of ligands onto material surfaces for the study of cell receptor spacing and cell–material interactions. Topography can be introduced on material surfaces, alongside biochemical ligand patterning, thereby defining the microenvironment. Engineering multi-peptide complexes enables binding of several receptors, bringing them in close association. DNA origami provides a tool for precise ligand presentation and hence the study of receptor clustering. Tension sensors enable the study of the downstream signaling that is associated with force generation following receptor binding. All of these techniques enable the study of the nanoscale receptor spatial organization and the downstream effects on cell fate when interfaced with a biomaterial

The material-based research that underpins regenerative medicine encompasses the fields of tissue engineering and nano-therapeutics. Tissue engineering aims to develop treatments for specific tissue defects by providing a scaffold that replicates or exploits the structural and spatiotemporal signaling complexity of the tissue microenvironment, thereby providing a platform that supports cell integration and ECM formation. While nano-therapeutics utilize nanoparticles, decorated with functional groups, binding domains, or growth factors, to detect and target specific cell surfaces or molecules to induce local responses [71–74]. Here, we discuss the advances in material fabrication techniques that are used to incorporate bioactive molecules onto the surfaces of materials (Fig. 2), in order to improve our understanding of material–cell signaling at the nanoscale that may influence tissue development, immunity, and repair.

### Hydrogels

Hydrogels are comprised of hydrophilic polymer chains, connected by physical or chemical cross-links, dispersed in a liquid medium. Although not a method of functionalization, hydrogels represent a class of highly tunable materials that have facilitated numerous studies of cell–material interactions. Hydrogels can be functionalized with specific cell-binding and proteolytic sites via chemical conjugation of peptides using a variety of coupling procedures [75]. The cell adhesion peptide RGD, derived from fibronectin, is most commonly used to impart or improve the cell-binding properties of hydrogels [76], while collagenase [77] or matrix metalloproteinase (MMP) [78–81] cleavable peptides are most commonly added to impart or increase proteolytic sites in hydrogels. The ability to functionalize hydrogels in a controlled manner has led to a number of findings that have advanced our understanding of cell–material interactions. Generally, the inclusion of RGD peptides has been shown to influence cell viability [82, 83], differentiation [84], and expression [85], while the inclusion of MMP peptides has been shown to promote outward cell migration [80]. Specifically, stretchable poly(N-acryloyl glycinamide)-based hydrogel systems have been used to alter ligand spacing in a single direction and demonstrate that osteoblasts and fibroblasts are unable to stably adhere to hydrogel surfaces when the distance between neighboring adhesion ligands is > 70 nm in one direction even if the ligand spacing between neighboring ligands in the opposite direction is ≤ 70 nm [86]. Poly(ethylene glycol) (PEG) diacrylate hydrogels, functionalized with PEG spacers of increasing length, have been used to show that increasing the distance of RGD peptides from the hydrogel surface decreases the concentration of RGD required to support corneal epithelial cell attachment and spreading [87]. Furthermore, by varying both the surface density and spatial distribution of RGD on the surface of poly(ethylene oxide)-based hydrogel, it has been shown that fibroblast migration speed is a function of surface ligand density and that clustering ligands reduces the ligand density required to support cell migration [88].

### Chemical Patterning

Cell signaling is dictated, in part, by chemical mediators in the immediate environment, which can be exploited in studying and manipulating cell signaling. Microscale chemical patterning has been in use since the 1990s. Micro-contact printing can be used to stamp specific molecules of defined shapes and sizes onto a surface, and therefore facilitates the study and control of cell binding and spreading [89]. The use of this technique has demonstrated that the shape of the adhesive islands can affect cell survival and differentiation [90–92]. For example, in the study by Chen and colleagues [90], endothelial cells grew and spread on 20 μm fibronectin (FN) islands; however, on 10 μm islands, cell death significantly increased. Von Erlach and colleagues [92] produced triangular, square, and circular FN micropatterns of the same surface area (1350 μm^2^) directing high to low cytoskeletal contractility and osteogenic to adipogenic differentiation of MSCs, respectively. In turn, disrupting the nanoscale arrangement of lipid rafts in the plasma membrane abolished the shape-induced differences in MSC contractility and differentiation. Thus, the chemical composition and shape of the ligand patterned area on a material as well as the nanoscale organization of membrane components induced by chemical mediators are important factors to consider when designing cell-instructive materials that support cell proliferation and differentiation. Soon after the introduction of micro-contact printing, dip-pen nanolithography was developed, enabling multiplexed deposition of molecules via an atomic force microscopy tip with nanoscale precision in a positive printing mode [93]. This technique has also been used to measure single molecular interactions, e.g., between integrin αvβ3 and vitronectin [94].

### Topographical Patterning

3D material topography is an additional characteristic that has been exploited to direct cell signaling in numerous studies. Chemical etching of silicon wafers can form precise relief patterns, reverse masks, or can be used to produce nanoscale structures that are directly seeded with cells. The use of a silicon mask to produce grooves in a polydimethylsiloxane (PDMS) mold has demonstrated that constraining cells within grooves alters their epigenetic markers, enabling reprogramming of cells [95, 96]. In another instance, porous silicon nanoneedle structures have delivered multiple payloads (nanoparticles, proteins, and nucleic acids) [97–99], in addition to monitoring the tissue pH environment, thereby enabling both delivery and diagnostics [100].

Combining specific 3D surface topography with chemical functionalization can improve the biocompatibility of materials for clinical applications. One such study combined reactive chemistry with surface micropatterning by spin coating a cyanoacrylate tissue glue onto polycaprolactone (PCL) patches that were previously patterned via hot embossing onto an ion-etched silicon wafer. Results showed that the quantity of cyanoacrylate tissue glue that is required to achieve tissue adhesion is reduced on micropatterned patches compared with flat patches [101].

Taking into account nanoscale spatiotemporal chemical profiles, such as cell surface receptor spacing, along with the microenvironment topography could aid the fine-tuning of materials to minimize off-target effects on cell signaling.

### Peptide Self-Assembly

Self-assembly enables the organization of complex biological structures and is used to precisely synthesize stimuli-responsive, complex nano-materials for more precise drug delivery and tissue repair. Nanostructures comprised of multiple binding peptides can be used to induce clustering of receptors and hence modulate cellular activity. These structures are designed bio-mimetically and can incorporate several different peptides together in a complex that is capable of simultaneously clustering multiple proteins or temporally releasing factors [102]. Different types of self-assembling peptides have been used for regenerative medicine applications. Fiber-forming coiled-coil–based peptides that assemble to display carbohydrate and ligands have been used for antigen ligand display [103]. The peptide RADA16-I-BMHP1 has been mixed with poly(lactic-co-glycolic acid) nano-fibers for functional nerve regeneration [104]. The self-assembling peptide hydrogel SPG-178-Gel has been used for bone regeneration [105], and an arginine-rich peptide has been used for the delivery of nucleic acid therapeutics [106]. This approach allows materials to interact with protein complexes on the cell surface and elucidate the cell dependence on precise spatiotemporal presentation.

### DNA Origami

DNA origami is a method used to control receptor positioning, whereby DNA is built in 2D or 3D and functionalized with chemical moieties at defined locations. This approach enables a high degree of spatial control, up to 5 nm [107], and is therefore effective in controlling the presentation of ligands to cells. Ligand nano-calipers have been used to arrange DNA origami modified with ephrin ligands to define EphA2 receptor spatial distribution and receptor-mediated signaling [108]. Precise nano-patterning of antigens using DNA origami has demonstrated that the binding affinities of antibodies change with antigen distances, with a distinct preference observed for antigens separated by approximately 16 nm [109]. These antigen patterns have implications for stimulating immune responses, whereby changing the specific distances of certain antigens could increase the efficacy of vaccinations or decrease an immune response to biomaterials. DNA origami has also been used to fabricate biomimetic nanoarrays enabling multivalent analysis of ligand–receptor interactions with nanoscale spatial resolution [110]. In this way, DNA origami can be utilized as a tool to probe the effects of spacing on receptor signaling. DNA origami could be incorporated into nanoparticle fabrication so that chemical moieties are presented to cells at defined distances.

### Tension-Mediated Sensing

Functionalizing material surfaces with biomolecule sensors that measure tension or allow movement of molecules permits mechanical studies at the molecular level to elucidate how tension-mediated signals are experienced by the cell. Often, tension sensors have a FRET-based read-out (determined by the proximity of two fluorescent molecules), which is reversed upon higher tension. DNA nanoparticle tension sensors have been utilized to measure integrin receptor tension during cell adhesion [111] and to demonstrate that T cell receptors transmit defined forces to their antigens, thus showing that the cells can optimize their specificity to defined ligands [112]. Hence, ligand spacing and affinity can further alter cell behavior in response to defined forces, so combining spatial presentation of bioactive molecules alongside material stiffness should be considered in the design of engineered biomaterials.

## Nanoscale Imaging of Cell Surface Receptor Organization and Cell–Material Interactions

The application of precisely defined fabrication techniques has revolutionized the construction of synthetic biomaterials that replicate the nanoscale organization and presentation of adhesive and cell-instructive ligands present in tissue. Traditionally, ligand display was inferred from the design of surface modifications and monitoring of the cell response including adhesion, cell spreading, and the formation of focal adhesions which could be monitored using confocal microscopy [41, 113, 114]. However, advances in nanoscale imaging have enabled material ligand spacing, cell adhesion receptors, and their interactions at the cell–material interface to be investigated with improved precision. In this section, we critically discuss applications of key nanoscale imaging characterization tools. We compare the relative strengths and weaknesses in these analytical tools in Table 1 and provide some prospective regarding how these might be applied to optimize next-generation biomaterial design.

**Table 1:** Strengths and weaknesses of analytical tools for nanoscale cell-material characterization

	Technique	Sample type			Resolution	Imaging thickness	Quantitative	Live imaging	Label free	Limitations	Future applications	References
		Cells	Material	Cell–material								
Diffraction limited fluorescence microscopy	Confocal microscopy	✔	✔	✔	200 nm (XY), 500 nm (Z)	200 μm	Semi (diffraction limited)	✔	✖	-Photo-damage to cells, particularly when high laser power is required in weakly labeled samples. -Poor light penetration can limit observation of cell–material responses particularly in dense, opaque, or thick biomaterial systems. Multi-photon and light-sheet microscopy have better optical sectioning capabilities than confocal microscopy.	-Fluorescence cross-correlation spectroscopy (FCCS) and image correlation spectroscopy (ICS) have been used to study the molecular dynamics of fluorescently labeled proteins in living cells. Future applications could apply these powerful approaches to develop further understanding of the molecular dynamics of these adhesion proteins and proteins associated with their assembly.	[40, 41, 115–121]
Super-resolution fluorescence microscopy	Single-molecule localization microscopy (SMLM)	✔	✔	✖	20–50 nm (XY), 100 nm (Z)	1–5 μm	✔	✔	✖	-Both STORM and PALM require specific fluorophores. Photo-switching tags for STORM (e.g., Alexa Fluor 647, Cy3, Cy5), while PALM requires photo-switchable or photo-activatable proteins (e.g., photo-activatable GFP). -STORM and PALM require extensive optimization, including high labeling density (to obey the Nyquist–Shannon theorem) and the use of oxygen scavenging buffers in STORM.	-SMLM approaches are yet to be optimized to evaluate adhesive-functionalized hydrogels and the interactions which exist at the cell–material interface.	[12, 114, 122–125]
	Structured illumination microscopy (SIM)	✔	✔	✔	100 nm (XY), 250 nm (Z)	< 15 μm	✔	✔	✖	-To exploit the Moiré effect for super-resolution imaging, at least three rotating wide-field images per image slice in the Z-stack. Dyes must therefore demonstrate good photostability throughout image acquisition in order for effective image reconstruction without artifacts.	-Thick or dense biomaterials scatter light extensively. These may present challenges to imaging. Newer techniques including instant two-photon SIM or light-sheet SIM are being reported, but they are yet to be applied specifically to study the cell–material interface.	[38, 126–129]
Electron microscopy	Scanning electron microscopy (SEM) and Focused ion beam (FIB)-SEM	✔	✔	✔	< 5 nm (XY), 50 nm (Z)	-	✖	✖	✖	-Very time- and labor-intensive sample preparation. Can be subject to artifacts due to sample dehydration, processing, and staining. -More difficult to investigate dynamic processes which may be possible to observe using live-cell imaging techniques that are available using fluorescence imaging or Raman spectroscopy.	-Correlative light and electron microscopy or correlation of EM techniques to nanoscale secondary ion mass spectrometry (NanoSIMS) imaging could reveal new insights when combined together in the context of the cell–material interface.	[130]
Physical	Atomic force microscopy (AFM)	✔	✔	✔	< 1 nm (XY)	< 20 nm	✔	✔	✔	-Single-scan images achievable are around 150 μm^2^, which is more restrictive than EM techniques. -Thermal drift can be problematic, particularly for long imaging times.	-Combining AFM with optical fiber nanospectroscopy (e.g., tip-enhanced Raman Spectroscopy) enables precise spectroscopic assessments of samples enabling important spatial and compositional information to be resolved.	[131–134]
Secondary ion mass spectrometry	Time of Flight (ToF)-SIMS	✔	✔	✔	200 nm (XY), < 1 nm (Z)	Static SIMS < 5 nm	✖	✖	✔	-Static SIMS is capable of analyzing the surface of biomaterials and therefore ideal for monitoring surface treatments. Depth profiling is possible with dynamic SIMS, but the depth of penetration is challenging to control. -Material analysis conducted under ultra-high vacuum.	-Further enhancements in the mass resolving power with the OrbiTrap mass analyzer in Orbi-SIMS offer new potential to study in detail the metabolomic influences that functionalized biomaterials may have on cell response and behavior.	[135–137]
	NanoSIMS	✔	✖	✖	50 nm (XY), < 1 nm (Z)	< 5 nm	✖	✖	✔	-Very limited number of instruments available globally. -Unlike ToF-SIMS, NanoSIMS is limited to the detection of elements or small fragments such as CN-. Isotope labeling of biomolecules is therefore key to detecting biomolecules of interest with NanoSIMS.	-NanoSIMS imaging could be combined with other SMLM optical approaches in order to validate the resolution of the technique. Significant need to expand NanoSIMS to characterize material functionalization.	[138]
Vibrational spectroscopy	Raman	✔	✔	✔	250 nm (XY)	50 μm	Semi	✔	✔	-Raman scattering is inefficient, requiring prolonged image acquisition times and excessive sample exposure to laser power which can induce photo-toxic effects in cells. Newer Raman spectroscopy approaches may reduce acquisition times including Coherent anti-Stokes Raman spectroscopy (CARS) or Surface-enhanced Raman spectroscopy (SERS) by boosting signal intensities.	-Given that Raman spectroscopy can be conducted on live-cell samples, there remains a need to characterize spatiotemporal interactions of cells with functionalized biomaterial systems using this technique.	[139, 140]

### Fluorescent Imaging

Due to the Abbe/Rayleigh limit of light diffraction, conventional fluorescence microscopes have a resolution limit of 200 nm, which prevents accurate nanoscale imaging of adhesion receptor clustering and other molecular cell processes. To circumvent the diffraction limit, single-molecule localization microscopy (SMLM) techniques, such as stochastic optical reconstruction microscopy (STORM) [141] and photo-activated localization microscopy (PALM) [142], have emerged.

SMLM has contributed to our current understanding of the organization of cell surface receptors, revealing the intricate details of their clustering, the assembly of accessory proteins, and downstream cell signaling in response to focal adhesion formation [48, 143–145]. Furthermore, nanoscale ligand display from biomaterials can be visualized and quantified with near to single-molecule accuracy. For example, RGD ligand nano-domains spaced 52 nm apart were imaged on thin films of co-polymers of polystyrene and poly(ethylene oxide) using STORM [114].

Despite these advances, achieving single-molecule resolution is a major challenge in cells interfaced with functionalized biomaterials. The complex image acquisition and processing necessary to analyze fluorophore photo-activation or photo-switching in thicker samples are often problematical. To bypass these technical issues, alternative optical methods have been employed for biomaterial investigation, albeit with compromised spatial resolution, including 3D-structured illumination microscopy (3D-SIM).

Instead of relying on fluorophore properties, 3D-SIM achieves resolution enhancement through software-mediated extraction of high-frequency information from rotating wide-field fluorescence images [126]. Compared with SMLM, 3D-SIM requires less fluorophore-labeling optimization, and specific imaging buffers are not necessary. The trade-off is that 3D-SIM fails to resolve single molecules, imaging protein clusters at best, with maximal lateral resolution of 100 nm. Despite such a limitation, 3D-SIM has directly linked ligand patterning with cell responses at the nanoscale. It is now possible to correlate the dynamic spatial organization of RGD ligand cluster growth on a biomaterial surface with the assembly of cellular focal adhesions [127].

### Electron Microscopy

Electron microscopy (EM) provides nanometer-level resolution, which can interrogate ultra-structural details of cell organelles, complex biomaterials, and the physical interactions that exist at the cell–material interface [146]. Combining gold immunolabeling with EM has enabled visualization of biochemical information at the nanoscale. For example, combining immuno-gold labeling and scanning EM (SEM), the redistribution of αvβ3 and αvβ1 integrin receptors in the membrane of MSCs seeded onto RGD-coated gold nanorods of varying aspect ratio was visualized, illustrating how bioactive ligands can regulate cell behaviors and the use of nano-engineered platforms in understanding fundamental cell mechanisms [147].

Furthermore, combining immuno-gold labeling with the 3D reconstruction capabilities of focused ion beam-scanning electron microscopy (FIB-SEM), an appreciation of the intricacy of 3D material physical topography and its influence on the biochemical signaling of cellular uptake mechanisms could be deciphered [97]. In an additional study, cells seeded on microgroove substrates were evaluated using immuno-gold FIB-SEM to correlate the morphological changes in the cell with the redistribution of histone marker H3K9me3 to the nuclear laminar and periphery of the cell [130].

### Atomic Force Microscopy

Since its first discovery over 30 years ago, atomic force microscopy (AFM) has made significant contributions to the characterization of bio-interfaces. Today, the variety of different AFM modes available can spatiotemporally map topographical, mechanical, electrostatic, and binding site functionality present on the surfaces of materials and cells with unprecedented atomic length–scale resolution [131].

Different AFM modes can be used to explore material topography, with the most widely applied including (1) contact-mode AFM, where the cantilever deflection is kept constant by adjusting the distance between the stylus and sample, or (2) dynamic-mode AFM, where the cantilever is oscillated and dynamically interacts with the surface of a material. Furthermore, by functionalizing the AFM tip, it is possible to resolve the organization of integrin-binding nanopatterns formed by DNA origami [110], gold nanoparticles [148], and dendrimers [149].

Further adaptations to the AFM instrumentation allow the quantification of substantially more complex interactions residing between cells and at the cell–material interface [132]. In single-cell force spectroscopy (SCFS), single cells are attached to tip-less AFM cantilevers, which have been coated with positively charged cell–adhesive polymers, such as poly-L-lysine. Under physiological conditions, single cells can be brought into close contact with the other cells or materials for a specified time and then removed while time and force curves are generated to quantify adhesive interactions [132].

This pioneering approach was first used to quantify cell–cell adhesion between trophoblasts and uterine epithelial cells [150]. Since then, substantial enhancements to SCFS have been developed, with availability of commercial AFM instruments capable of enhanced pulling ranges (> 100 μm), precision (0.1 nm), and force sensitivity (5 pN) [151]. These optimized setups enable SCFS to probe adhesion interactions over a much broader range of detachment forces. Recently, SCFS proved that human neural stem cell de-adhesion was largely driven by the discrete unbinding of integrin–RGD complexes as opposed to being mediated by elastic restoration of gelatin methacrylate (GelMA) chains [152], while early co-operation between α2β1-mediated adhesion receptors on nanopatterned collagen type I matrices was critical in order to form higher-order adhesion structures [153]. By careful experimental design, AFM can be used to specifically link receptor organization to biophysical effects, which could be used to quantitatively optimize adhesive biomaterial design.

### Nanoscale Secondary Ion Mass Spectrometry

In nanoscale secondary ion mass spectrometry (NanoSIMS), a high-energy primary Cs^+^ or O^−^ beam dynamically splutters secondary ions from thin sections to reveal metabolic and compositional information about biological materials. This enables isotopically tagged proteins and small molecules within cells to be imaged at lateral resolutions of around 50 nm [138].

NanoSIMS analysis has been performed to identify specific cell receptors, with one detection approach making use of fluorinated nanobodies [154]. These are smaller and less prone to aggregation than gold-labeled antibodies traditionally used in EM analysis and have been used to show that T cell receptor aggregation occurs in response to major histone compatibility (MHC) complex activation, where the T cell receptors were found to cluster between 60 and 150 nm in the plasma membrane [155].

Moreover, NanoSIMS analysis can spatially resolve small-molecule metabolite distributions. For example, ^14^N-sphingomyelin precursors were shown to organize in specific membrane regions, called lipid rafts [156]. These lipid raft regions often control cell shape and play a pivotal role in the positioning and organization of cell surface receptors [157, 158].

NanoSIMS provides strong multiplexing capabilities. This was demonstrated using lanthanide-based immunohistochemistry, where it was possible to resolve the location of 10 unique proteins in breast tumor sections [159]. Such capabilities are to date unmatched by other nanoscale techniques such as EM.

Despite these advantages, the specialist nature of this technique and the limited instrumentation globally have so far restricted the use of NanoSIMS analysis in the study of cell–biomaterial interactions. The ability to correlate NanoSIMS analysis with SMLM and EM imaging offers great potential for nanoscale analysis of cells and the cell–biomaterial interactions.

## Future Perspectives

This review paper highlights a number of important insights that we hope will be useful for the field of regenerative medicine. Firstly, our understanding of how cells interpret differences in ligand presentation must be improved in order for subtle differences to be exploited in precision medicines. Secondly, a variety of material-based techniques are available to better understand the molecular biology underlying ligand positioning in biological systems. Thirdly, characterization of the interface between biomaterials and cells at the nanoscale must be performed, with the techniques capable of achieving this resolution discussed herein. The techniques discussed here are by no means exhaustive, but indicate a large breadth of methods that currently exist to probe various nanoscale cellular mechanisms and functionalize biomaterial surfaces. This combined effort could lead to more precise regenerative medicine strategies that can be effective at lower doses with decreased off-target effects.
